# Posttraumatic Lymphedema after Open Fractures of the Lower Extremity—A Retrospective Cohort Analysis

**DOI:** 10.3390/jpm11111077

**Published:** 2021-10-24

**Authors:** Johannes Maximilian Wagner, Victoria Grolewski, Felix Reinkemeier, Marius Drysch, Sonja Verena Schmidt, Mehran Dadras, Julika Huber, Christoph Wallner, Alexander Sogorski, Maxi von Glinski, Thomas A. Schildhauer, Marcus Lehnhardt, Björn Behr

**Affiliations:** 1 Department of Plastic Surgery, BG-University Hospital Bergmannsheil, 44789 Bochum, Germany; max.jay.wagner@gmail.com (J.M.W.); strohvictoria@gmail.com (V.G.); Felix.Reinkemeier@ruhr-uni-bochum.de (F.R.); Marius.Drysch@ruhr-uni-bochum.de (M.D.); sva.schmidt@yahoo.de (S.V.S.); mdadras@outlook.com (M.D.); julika.huber@hotmail.com (J.H.); c.wallner88@gmail.com (C.W.); alexander.sogorski@gmx.de (A.S.); maxi.vonglinski@bergmannsheil.de (M.v.G.); marcus.lehnhardt@rub.de (M.L.); 2 Department of Traumatology and Orthopedic Surgery, BG-University Hospital Bergmannsheil, 44789 Bochum, Germany; thomas.schildhauer@bergmannsheil.de

**Keywords:** posttraumatic lymphedema, long bone fractures, soft tissue injury, lower extremity

## Abstract

Secondary lymphedema is a very common clinical issue with millions of patients suffering from pain, recurrent skin infections, and the constant need for a decongestive therapy. Well-established as a consequence of oncologic procedures, secondary lymphedema is also a well-known phenomenon after trauma. However, precise epidemiological data of lymphedema progress upon severe extremity injuries are still missing. In the present work, we analyzed a patient cohort of 94 individuals who suffered open fractures of the lower extremity and soft tissue injury, of 2nd and 3rd grade according to Tscherne classification, between 2013 and 2019. Typical symptoms of lymphedema have been obtained via interviews and patient medical records in a retrospective cohort analysis. Of all patients, 55% showed symptoms of secondary lymphedema and 14% reported recurrent skin infections, indicating severe lymphedema. Furthermore, comparing patients with and without lymphedema, additional parameters, such as obesity, total number of surgeries, infections, and compartment syndrome, related to lymphedema progress could be identified. According to these data, posttraumatic secondary lymphedema has a highly underestimated clinical prevalence. Further prospective studies are needed to validate this first observation and to identify high-risk groups in order to improve patient’s health care.

## 1. Introduction

Lymphedema is a localized fluid collection within the interstitium caused by insufficient lymphatic drainage, and is a progressive disease, being favored by two aspects: increased hydrostatic pressure, consequently pressing out more fluid into the interstitium and furthermore increased oncotic pressure, as lymph fluid is rich in protein [1]. Another contributing factor of lymphedema progression is recurrent infections of the skin. Lymph fluid is an ideal breeding ground for bacteria and even small skin lesions can lead to severe erysipelas. A rare but serious consequence after recurrent infections is lymphangiosarcoma [1,2,3]. According to its etiology, lymphedema can be classified as primary or secondary. Primary lymphedema is very rare and mostly caused by an innate dysfunction of lymph vessels. Most patients suffer from secondary lymphedema after tumor resection, radiation, infection, or overweight [1,3,4]. The most common cause of secondary lymphedema posed here is tumor resection. In particular, patients after cervical, axillary, or inguinal lymphadenectomy have a high risk of developing lymphedema [5]. Diagnosis of lymphedema is mostly based on clinical criteria (volumetry, circumferential measurements) and the presence of predispositional factors, such as malignant diseases or trauma. Additionally, ultrasound, CT, MRI, and especially near-infrared-fluorescence angiography are important tools to gain further clinical information about the severity and progress of lymphedema [6]. Important differential diagnoses of lymphedema are lipedema, chronical venous insufficiency, drug-induced edema, or hypoalbuminemia. 

A quite underestimated cause of secondary lymphedema is trauma. Patients suffer from secondary lymphedema especially after open or closed fractures of the long bones [7]. The accompanying lymphedema in the acute phase of bone and wound healing often leads to delayed wound healing and wound infections, requiring complex and prolonged treatments.

However, precise data of the prevalence of posttraumatic lymphedema are still missing. In the present study, we evaluated the prevalence of lymphedema in a patient cohort who suffered open fractures of the lower leg or thigh between 2013 and 2019, in a single institution. Furthermore, this work aimed to assess the prevalence of posttraumatic lymphedema in this patient cohort, and thereby identify patients who have a high risk of secondary lymphedema progress.

## 2. Materials and Methods

The study was conducted according to the guidelines of the Declaration of Helsinki, and approved by the Institutional Review Board of Ruhr University Bochum. In this work we performed a retrospective cohort study in order to assess the prevalence of posttraumatic lymphedema after open fractures of the lower leg or thigh, and to further identify risk factors of the occurrence of posttraumatic lymphedema. In total, 200 patients which suffered from open long bone fractures of the lower extremity with soft tissue injuries of grade 2 and 3 according to Tscherne, and were treated between 2013 and 2019 in a single institution were identified and contacted personally via telephone and asked about prolonged swelling of the injured extremity, need for compression garments, manual lymph drainage, pain, and recurrent skin infections.

Soft tissue injury of patients was classified according to ICD-10, adapted from Tscherne and Oestern classification.

Moreover, clinical, demographic, and outcome data were assessed from medical records. In total, 106 of these patients could not be contacted successfully and had to be excluded from the analyzed patient cohort.

The prevalence of lymphedema was defined by the existence of at least a prolonged swelling and the constant need for a decongestive therapy. Furthermore, references for a secondary lymphedema had to be found within the medical records, which were based on a prolonged swelling of the affected limb, the occurrence of pitting edema, or stemmer sign.

### 2.1. Patients

Inclusion criteria for this study were long bone fractures (femur, tibia, fibula) of the thigh and lower leg combined with soft tissue injuries grade 2 and 3, according to Tscherne classification, receiving open reduction, and fixation in a single institution between 2013 and 2019.

### 2.2. Statistics

Data are shown as means (range) or median. Patients were subdivided into two groups according to the occurrence of lymphedema and non-lymphedema. Pearson’s chi-square test and Fisher’s exact test was used for categorial variables (when expected value of any cell was below 5, Fisher’s exact test was used instead of chi-square test). For continuous variables, independent t-test was used. *p*-value below 0.05 was considered statistically significant.

## 3. Results

A total of 70 men and 24 women who suffered from open long bone fractures and 2–3° soft tissue injury between 2013 and 2019 were contacted personally via telephone. According to specific symptoms and additional information based on their medical records, a secondary lymphedema could be found in 52 (55%) of these 94 patients. Thirteen of these patients (14%) reported recurrent skin infections, indicating a severe lymphedema of the injured extremity.

Patient related characteristics are shown in Table 1. Although about three times more men than women existed in the analyzed patient cohort, no gender specific differences between the lymphedema and non-lymphedema group could be noted. 

The mean age of all patients included was 48.6 years (16–84 years). The mean age of patients with lymphedema (48.48 years) did not differ from patients without lymphedema (48.92 years, *p* = 0.904). Interestingly, when comparing different age groups in detail, a significantly increased number of patients in the lymphedema group were noted between 40 and 65 years (61.5% vs. 35.7%, *p* = 0.013).

Furthermore, significantly more patients with severe obesity (BMI > 30 kg/m^2^) (BMI upon trauma) suffered from posttraumatic lymphedema (19.2% vs. 4.8%, *p* = 0.035). No differences between both groups could be found concerning pre-existing conditions, such as arterial hypertension and diabetes mellitus type 2. Of great interest was the fact that significantly more patients with lymphedema (63.5%) suffered from pain in the injured extremity than patients without lymphedema (30.9%, *p* = 0.001).

As there were few differences in patient characteristics related to the occurrence of posttraumatic lymphedema, we wondered if characteristics of the trauma itself, treatment, and complications could be helpful to find distinct variables related to lymphedema. 

Trauma related characteristics are presented in Table 2. 

The vast majority of all patients suffered fractures of the tibia (97.9%), while femur fractures could only be noted in 7.4%. Furthermore, a slight majority of 67.3% of lymphedema patients showed multiple fractures of the lower extremity, however, compared to non-lymphedema group (52.4%), no statistical difference became evident (*p* = 0.141).

Comparing different mechanisms of injury in our patient cohort, we categorized contusion, traffic accident, and fall. Interestingly, a significantly increased number of patients in the lymphedema group (53.9%) sustained a traffic accident compared to trauma patients without lymphedema (28.6%, *p* = 0.013). Soft tissue injury, according to Tscherne classification (Table 3), was evenly distributed in lymphedema and non-lymphedema group. 

Interestingly, vascular trauma and polytrauma patients were not significantly enhanced in the lymphedema group (*p* = 0.099; *p* = 0.138), although a distinct trend could be noted in vascular trauma patients developing a lymphedema.

Having analyzed trauma characteristics of the patients, we were further interested in treatment and complications as important indicators for lymphedema progress. Subsequently, we matched total number of surgeries, which indirectly exhibited the severity of trauma and soft tissue injury. Interestingly, only 1.9% of lymphedema patients required one or two surgeries, while 31% of non-lymphedema patients could be successfully treated with this small number of interventions. Most lymphedema patients needed five or more surgical interventions (67.3%), while this quantity was significantly smaller in non-lymphedema patients (40.5%, *p* = 0.009). Furthermore, significantly heightened number of lymphedema patients required skin grafts (57.7%, *p* = 0.018) or flap tissue reconstruction (53.8%, *p* = 0.014).

Finally, infections of soft tissue or bone, as well as the occurrence of a compartment syndrome of the lower leg seemed to be related to lymphedema development in trauma patients (*p* = 0.020; *p* = 0.035).

## 4. Discussion

Although posttraumatic lymphedema is a well-known problem of orthopedic surgery, there is still a lack of precise epidemiological data in current literature. First descriptions on the occurrence of secondary lymphedema after trauma to the affected limb can be found in Italian and Russian literature in the 1960s [8,9]. 

In the present study, we analyzed 94 patients suffering from traumatic fractures and 2° and 3° soft tissue injury of the lower limb who were treated at a single institution between 2013 and 2019. Although these patients are believed to be at high risk of developing secondary lymphedema [7], the data presented here indicate that the occurrence of posttraumatic lymphedema is a highly underestimated issue. More than half of the analyzed patients showed typical symptoms of a secondary lymphedema of the injured limb. This prevalence number is comparable to other patients who are at a high risk of secondary lymphedema, for example breast cancer patients after complete axillary lymph node dissection with a prevalence of 20–50% [6]. As already mentioned previously, relevant literature providing epidemiological data is still rare. Only few studies reported about the occurrence of this highly relevant issue in trauma patients. For example, Pereira et al. performed SCIP lymphatic vessel transfer in patients with secondary lymphedema after trauma, and furthermore were able to surgically prevent development of secondary lymphedema after soft tissue injury in a small patient cohort [10,11]. However, no information was given about the prevalence of secondary lymphedema. Furthermore, successful treatment of prolonged hand lymphedema after trauma was reported in two young woman by vascularized lymph node transfer [12]. A rare but very serious complication of a prolonged lymphedema, the occurrence of an angiosarcoma, has been reported by Trattner et al. as a consequence of posttraumatic lymphedema [13]. 

While there is certain evidence for the appearance of a posttraumatic lymphedema, it can only be speculated about the underlying pathomechanism. Interestingly, Szczesny et al. reported about dilatated lymph vessels and lymph nodes during the follow-up of patients with lower extremity fractures and assumed an ongoing inflammatory process, contributing to the development of a secondary lymphedema after trauma [14]. An obvious explanation in our case series would be the disruption and lack of proper regeneration of the lymphatic system at the injury site.

After defining the prevalence of posttraumatic lymphedema in this particular patient cohort, which is demonstrably at high risk, we were interested in further patient or trauma related properties that could be used to define high-risk groups.

Interestingly, only limited findings could be concluded from patient characteristics. While obesity seems to contribute to lymphedema progress in this context, patients’ age only had little impact. Although significant differences could be detected in the age group of 40 years to 65 years, we assume this result to be biased by an increased severity of trauma within this group. Moreover, we propose that the severity of trauma is directly related to occurrence of lymphedema. However, which parameters should be taken into account for this purpose? We assumed that the grade of soft tissue injury directly affects the incidence of posttraumatic lymphedema, which could be concluded from oncologic studies, examining the occurrence of secondary lymphedema after extremity sarcoma resection. According to Wu et al., patients with large tumors at the medial thigh have the highest risk of secondary lymphedema after tumor resection [15].

To our surprise, soft tissue injury classification according to Tscherne did not show significant differences in the occurrence of a lymphedema, and therefore seemed not to be suitable, similar to the assessment of multiple fractures or polytrauma.

The most sensitive parameter which could be identified in the data analysis was the number of total surgeries. Only one patient requiring two surgeries developed a lymphedema, while 31% of patients in the non-lymphedema group with one or two surgical interventions was free of lymphedema symptoms. The authors assume the number of surgeries to be a sensitive parameter for trauma severity, as patients with highly traumatized soft tissue often require multiple surgeries.

We provide first data reporting the prevalence of secondary posttraumatic lymphedema. However, these data are preliminary and need to be further validated by prospective studies. Furthermore, classification of soft tissue injury has been performed by different surgeons, which could have caused a possible bias and has to be taken into account when interpreting these data. 

Further limitations of this work concern the retrospective lymphedema assessment based on telephone interviews and the corresponding medical records. 

## 5. Conclusions

The analysis of this patient cohort suffering from lower extremity fractures and soft tissue injury did reveal a very high prevalence of secondary lymphedema, even multiple years after trauma. The results of this study suggest that the appearance of a posttraumatic lymphedema seems to be a highly underestimated clinical issue. Although the first interesting parameters for identification of high-risk groups could be identified, further studies are mandatory to identify patient cohorts who are at highest risk of lymphedema development. This may help to reduce the burden of chronic lymphedema for these patients or could even help to prevent the occurrence or enable early interventions to decrease symptoms.

## Figures and Tables

**Table 1:** Patient characteristics.

	All Patients *n* = 94 (%)	Patients with Lymphedema *n* = 52 (%)	Patients without Lymphedema *n* = 42 (%)	*p*-Value (Comparison)
Age mean (median)	48.6 (16–84)	48.48 (17–84)	48.92 (16–80)	0.904
>40	30 (31.9)	14 (26.9)	16 (38.1)	0.248
40–65	47 (50.0)	32 (61.5)	15 (35.7)	**0.013**
>65	17 (18.1)	6 (11.5)	11 (26.2)	0.067
Sex				
Male	70 (74.5)	42 (80.8)	28 (66.7)	0.119
Female	24 (25.5)	10 (19.2)	14 (33.3)	
Obesity (BMI > 30 kg/m^2^)	12 (10.6)	10 (19.2)	2 (4.8)	**0.035**
Diabetes mellitus type 2	10 (10.6)	6 (11.5)	4 (9.5)	0.106
Arterial hypertension	29 (30.9)	16 (30.8)	13 (31.0)	0.309
Pain	46 (48.9)	33 (63.5)	13 (30.9)	**0.001**

**Table 2:** Trauma characteristics.

	All Patients *n* = 94 (%)	Patients with Lymphedema *n* = 52 (%)	Patients without Lymphedema *n* = 42 (%)	*p*-Value (Comparison)
Trauma				
Contusion	27 (28.7)	13 (25.0)	14 (33.3)	0.375
Traffic accident	40 (42.6)	28 (53.9)	12 (28.6)	**0.013**
Fall	27 (28.7)	11 (21.1)	16 (28.1)	0.071
Soft tissue injury (Tscherne)				
2nd grade	34	17 (32.7)	17 (40.5)	0.362
3rd grade	60	35 (67.3)	25 (59.5)	0.435
Fractured long bone				
Femur	7 (7.4)	5 (9.6)	2 (4.8)	0.372
Tibia	92 (97.9)	50 (96.2)	42 (1.0)	0.199
Fibula	26 (27.7)	14 (26.9)	12 (28.6)	0.859
Total number of surgeries				
1	7 (7.4)	0 (0)	7 (16.7)	**0.003**
2	7 (7.4)	1 (1.9)	6 (14.3)	**0.042**
3	18 (19.1)	10 (19.2)	8 (19.0)	0.982
4	10 (10.6)	6 (11.5)	4 (9.5)	0.753
5 or more	52 (55.3)	35 (67.3)	17 (40.5)	**0.009**
Multiple fractures	57 (60.6)	35 (67.3)	22 (52.4)	0.141
Vascular trauma	7 (7.4)	6 (11.5)	1 (2.4)	0.099
Polytrauma	13 (13.8)	8 (15.4)	5 (11.9)	0.138
Infection (soft tissue, bone)	32 (34.0)	23 (44.2)	9 (21.4)	**0.02**
Compartment syndrome	12 (12.8)	10 (19.2)	2 (4.8)	**0.035**
Skin graft	44 (46.8)	30 (57.7)	14 (33.3)	**0.018**
Local, pedicled, free flaps	40 (42.6)	28 (53.8)	12 (28.6)	**0.014**

**Table 3:** Classification of open fractures according to Tscherne and Oestern 1982.

Grade	Soft Tissue Injury
1	minimal skin laceration
2	skin laceration, circumferential contusions, moderate contamination
3	extensive: major vascular and or nerve damage, compartment syndrome
4	subtotal and complete amputations

## Data Availability

Data sharing is not applicable to this article.
